# Eco-Friendly Photocatalytic Solutions: Synthesized TiO_2_ Nanoparticles in Cellulose Membranes for Enhanced Degradation of Indigo Carmine Dye

**DOI:** 10.1021/acsomega.4c04017

**Published:** 2024-10-15

**Authors:** Arthur Matsudo, Larissa V. F. Oliveira, Tereza S. Martins, Fernanda F. Camilo

**Affiliations:** 1 Chemistry Department, Institute of Environmental, Chemical and Pharmaceutical Sciences, Federal University of São Paulo, Diadema SP-09913-030, Brazil; 2 Center of Natural Sciences and Humanities, Federal University of ABC, Santo Andre SP-09210-580, Brazil

## Abstract

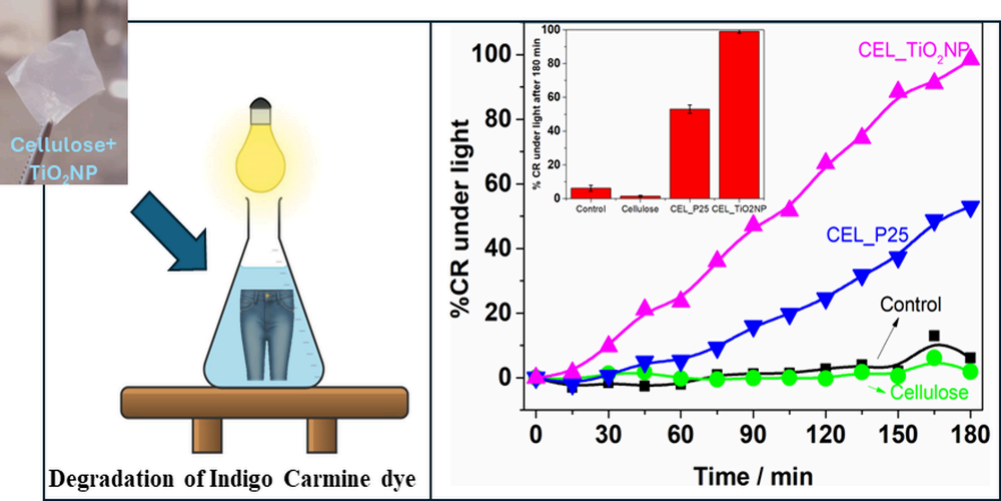

This study focuses on comparing the efficiency of commercially available TiO_2_ (P25) with synthesized TiO_2_ nanoparticles (TiO_2_NP) impregnated in nonmodified cellulose membranes, specifically targeting the degradation of Indigo Carmine (IC) dye. We developed a novel method to enhance the interaction between cellulose and TiO_2_, thereby improving efficiency and reusability. This involves dissolving microcrystalline cellulose in 1-butyl-3-methylimidazolium chloride (BMImCl) and dispersing the TiO_2_ samples within this solution. The resulting cellulose membrane embedded with TiO_2_ nanoparticles (TiO_2_NP) exhibited a higher adsorption capacity and greater photocatalytic efficiency against IC compared to that of P25. This improvement is attributed to the larger surface area and increased reactivity of the synthesized TiO_2_NPs. Furthermore, the CEL_TiO_2_NP membranes demonstrated excellent stability and reusability, maintaining their catalytic efficiency over multiple cycles. This study presents new opportunities for developing efficient, reusable photocatalytic materials for environmental remediation using eco-friendly cellulose.

## 1 Introduction

Titanium dioxide (TiO_2_) is a widely studied photocatalyst due to its low cost, nontoxicity, chemical and thermal stabilities, and effective dispersion in aqueous media. It has three main crystalline phases: rutile, anatase, and brookite. Rutile is the most common and easily formed phase upon heating either anatase or brookite, and the transformation is thermodynamically favorable and irreversible.^1−3^

The most well-known commercial TiO_2_ photocatalyst is P25 from Degussa, a mixture comprising 80% anatase phase and 20% rutile, with anatase noted for its superior photoactivity.^4−6^ The mechanism of TiO_2_ photocatalysis has been well described in the literature and centers on the creation of electron–hole pairs.^7,8^ When energy surpassing its bandgap is applied, electrons are excited to the conduction band (CB) and holes are created in the valence band (VB), facilitating surface reactions that reduce and oxidize oxygen and water molecules, respectively, generating superoxide reactive radicals (O_2_^**–.**^) and hydroxyl radicals (OH·) that can degrade organic pollutants.^9^Figure S1 (Supporting Information) presents a schematic representation of this mechanism.

Given the intrinsic nature of photocatalytic activity as a surface-dominated process, the use of TiO_2_ nanoparticles offers a critical advantage. These nanoparticles provide a vastly increased surface-to-volume ratio compared to their bulk material counterparts.^10^ This enhanced surface area significantly amplifies the photocatalytic efficiency, providing more active sites for photocatalytic reactions.

Building on the advantages of TiO_2_ nanoparticles, their incorporation into cellulose membranes marks a significant advancement in the field of heterogeneous photocatalysis.^11^ This innovative approach capitalizes on the benefits of cellulose as a biodegradable, renewable, and versatile substrate. Cellulose membranes, with their enhanced mechanical strength, flexibility, and resistance to dissolution in aqueous media, are crucial for heterogeneous catalysis. Furthermore, in contrast to powders or dispersions, cellulose membranes offer a more practical and environmentally friendly solution, especially useful in applications like water purification where ease of handling and reusability are key.^12,13^

In existing research, TiO_2_-cellulose hybrid materials have predominantly been explored in the powdered form or using cellulose derivatives for dye degradation.^14−16^ This trend highlights a significant opportunity to focus on the preparation of membranes using unmodified cellulose and TiO_2_. In this context, in 2012, in the study of Wu et al.^17^ titanium dioxide nanofibers were combined with cellulose fibers to create freestanding and flexible catalyst membranes. The photocatalytic activity of these anatase nanofibers, demonstrated in the decomposition of methyl orange dye in aqueous solutions, was found to be comparable to that of the well-known Degussa P25. Further progress was achieved in 2017 by Jiao et al.^18^ who developed a method to deposit anatase titania on cellulose paper. This hybrid paper, containing approximately 13.86 wt % of anatase TiO_2_, proved to be an eco-friendly photocatalyst that rapidly degraded blue Indigo Carmine dye into a colorless solution within 30 min under ultraviolet (UV) radiation. Later, Zhang and colleagues^19^ fabricated a transparent titanium dioxide (TiO_2_) @TiO_2_ freestanding film using cellulose paper as a template, which showed high photocatalytic activity for methylene blue degradation under UV irradiation. However, in these studies, no reuse tests were performed. Considering these developments, the search for methods to produce membranes of nonmodified cellulose containing TiO_2_ anatase nanoparticles remains an important and ongoing need in the field of photocatalysis, aiming to further enhance efficiency and practicality in various applications.

This study aims to compare the efficiency of commercially available TiO_2_ (P25) with synthesized TiO_2_ nanoparticles, particularly when integrated into nonmodified cellulose membranes, and to address challenges related to ease of use and reusability in photocatalytic applications, specifically targeting the degradation of Indigo Carmine dye. We attempt to overcome limitations identified in previous studies, where cellulose was used in the form of paper or fiber. This approach often leads to a heterogeneous distribution of particles and the potential for leakage, which can diminish the photocatalytic efficiency over multiple cycles. Our method involves dissolving cellulose in an ionic liquid, namely, 1-butyl-3-methylimidazolium chloride (BMImCl), in which the TiO_2_ samples are also dispersed. This technique aims to improve the interactions between organic and inorganic components, thereby potentially enhancing the photocatalyst’s effectiveness, especially in terms of reusability. Notably, this study utilizes inexpensive and benign microcrystalline cellulose, as opposed to derivative forms of cellulose.

## 2 Methods

### 2.1 Preparation of Titanium Dioxide Nanoparticles (TiO_2_NP)

Titanium dioxide nanoparticles were prepared following a procedure already described in the literature.^20^ Briefly, in a 250 mL glass beaker equipped with a magnetic stirrer, 2.50 mL (8.40 mmol) of titanium isopropoxide and 47.5 mL of isopropyl alcohol were added. The mixture was stirred constantly at room temperature until it was completely dissolved (SOLUTION A). In a 1000 mL round-bottom glass flask with magnetic stirring, 450 mL of ultrapure water (Milli-Q) and a few drops of HNO_3_ (65% m/m, Synth) were added to reach a pH of 1.5 (SOLUTION B). This mixture was kept in an ice bath until it reached 0 °C. Using an addition funnel, SOLUTION A was added to SOLUTION B at a rate of one drop per second. After the addition, the mixture was stirred for 24 h in an ice bath, becoming transparent. To obtain the nanoparticles in the powder form, the solvent was removed at 40 °C under reduced pressure (60 mmHg). When necessary, a nitrogen gas flow was used to remove the residual water. This sample was named TiO_2_NP.

### 2.2 Preparation of the Cellulose Membrane Containing Synthesized Titanium Dioxide Nanoparticles

Initially, 3.00 g of a cellulose dispersion (5.00 wt %; 150 mg of cellulose) dissolved in BMImCl was mixed with a dispersion containing 30.0 mg of TiO_2_NP dispersed in 2.00 g of BMImCl. This mixture was stirred for 30 min at room temperature. The mixture was poured onto a 12.0 cm diameter glass Petri dish, and a spin coater was used to spread it. The parameters used in the spin coater were: 120 s to reach 500 rpm, which was maintained for 40 s, followed by 10 s of deceleration. Then, water was added to promote the formation of the membrane, which was washed with water until the ionic liquid was completely removed. The complete elimination of BMImCl was verified by collecting aliquots of the washing water and treating them with an aqueous solution of AgNO_3_ (0.10 M). Afterward, the film was dried at room temperature for 24 h, resulting in a cellulose membrane named CEL_TiO_2_NP.

Another membrane was prepared following the identical procedure described above except for the use of commercial P25 from Degussa instead of the prepared TiO_2_ nanoparticles. This film was named CEL_P25.

### 2.3 Indigo Carmine Decolorization Tests Using TiO_2_ Nanoparticle Membranes (Commercial and Synthesized) under UV Light

The photocatalytic activities of the Cel_TiO_2_NP and Cel_P25 samples were evaluated by decolorizing an aqueous solution of Indigo Carmine (IC) by using ultraviolet–visible electromagnetic radiation. The light source used in the photocatalysis experiment was an ovoid-shaped Osram HQL 125W mercury vapor lamp^21^ fixed 10.0 cm above the samples at the top of the reactor. UV radiation was collimated using a light fixture to ensure that the same radiation intensity was received by all samples.

In the photocatalytic experiment, 40.0 mL of an IC solution (20.0 ppm) and 50.0 mg of each cellulose membrane with an initial pH of 2 (adjusted with HNO_3_) were placed in the reactor. The mixture was stirred in the dark for 60 min to adsorb the dye on the photocatalyst. Then, the flask was placed under UV–vis light, and aliquots were collected at defined time intervals. An experiment in the absence of a catalyst was performed for comparative purposes (control experiment). The decolorization of the solution was monitored using UV–vis absorption spectroscopy by tracking the decrease in the characteristic band of Indigo Carmine at 610 nm over time. In the reuse tests, the membrane was easily removed from the solution using tweezers, washed with distilled water, and then used in another test.

### 2.4 Equipment

X-ray diffractograms (XRD) were recorded on a BRUKER D8 DISCOVER diffractometer using a Cu Kα anode (0.1542 nm), operating at 40 kV and 30 mA. UV–vis absorption spectra of the TiO_2_NP dispersions were recorded on the OCEAN OPTICS USB 4000 spectrophotometer using a quartz cuvette with a 1.00 cm optical path. These spectra were obtained from a diluted dispersion (20.0 μL of sample: 2.00 mL of water). For photocatalytic tests, IC aliquots were recorded without dilution. Diffuse reflectance spectra (DRS) of the membranes were acquired using the same equipment with the samples positioned on a sample holder at a 90° angle relative to the light beam. Raman spectra were obtained on a RENISHAW brand Raman microscope, InVia model, equipped with a multichannel detector, with a He–Ne laser excitation at 632.8 nm and a diode laser at 830 nm, with a spectral resolution of 4.00 cm^–1^. Fourier-transform infrared (FTIR) spectra were recorded on a SHIMADZU IR-PRESTIGE-21 spectrophotometer, using an attenuated total reflection (ATR) accessory (ATR-8200HA) in the range of 400–4000 cm^–1^ with 4.00 cm^–1^ resolution. Thermogravimetric analyses (TGA) were recorded on a TA Instruments SDT 650 thermal analyzer, using a synthetic air atmosphere (100 mL.min^–1^), an alumina crucible (90 μL), and a heating rate of 10 °C.min^–1^ in the temperature range between 25 and 800 °C. Zeta potential and dynamic light scattering (DLS) were performed on the MALVERN Zetasizer Nano equipment. Scanning electron microscopy (SEM) images were recorded on a JEOL JSM-7401F microscope equipped with an energy-dispersive spectrometer (EDS). The samples were coated with a thin layer of gold to improve the electrical conduction of the samples and consequently the quality of the obtained image. Transmission electron microscopy (TEM) images were captured using a JEM 2100 microscope from JEOL, which was equipped with an energy-dispersive spectrometer (EDS). A droplet of the sample was placed onto a carbon-coated Cu microgrid and allowed to air-dry at room temperature. Inductively Coupled Plasma Optical Emission Spectrometry (ICP-OES) analyses were performed on a radial view ARCOS optical emission spectrometer from SPECTRO, using argon plasma and equipped with a solid-state charge-coupled device detector. The samples were digested using aqua regia and heated in a DigiPREP block digestion system from SCP SCIENCE.

## 3 Results and Discussion

**Figure 1:**
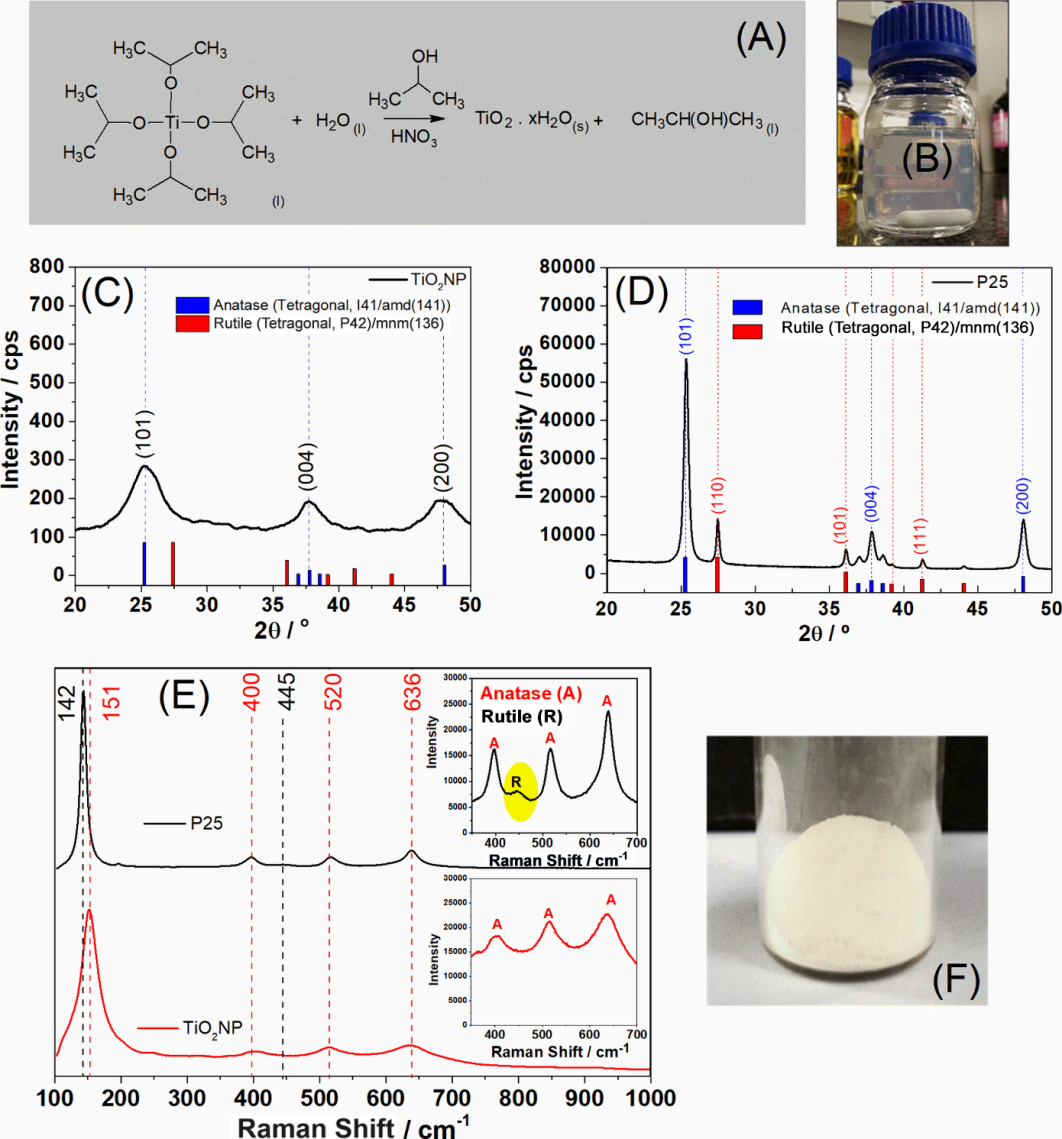
Chemical reaction for TiO_2_ nanoparticle preparation (A). Photograph of TiO_2_NP in dispersion (B). X-ray diffraction analysis of TiO_2_NP (C). X-ray diffraction analysis of P25 (D). Raman spectra of the TiO_2_NP and P25 (E). Photograph of TiO_2_ after the removal of water (F).

Titanium dioxide nanoparticles (TiO_2_NP) were synthesized through the slow hydrolysis of titanium(IV) isopropoxide in an acidic medium (Figure 1A). This process resulted in a slightly white dispersion without any precipitate formation at the bottom (Figure 1B). Upon removal of water, a white solid, denoted as TiO_2_NP was obtained (Figure 1F).

The crystalline phases present in the TiO_2_NP sample were analyzed by using X-ray diffraction (XRD) (Figure 1C). The diffractogram revealed peaks at 2θ values of 25, 37, and 48°, corresponding to the crystallographic planes (101), (004), and (200), respectively, of the anatase phase. This phase belongs to the tetragonal crystal system with space group I41/and (141) and is associated with titanium oxide (JCPDS N°. 21-1272). Notably, the peaks exhibited low intensity and broad nature, indicating a diminished crystallite size and low crystallinity. The P25 sample (Figure 1D) shows additional diffraction peaks at 27, 36, and 41°, corresponding to the crystallographic planes (110), (101), and (111) of the rutile phase, corresponding to the tetragonal crystal system with space group P42/mnm (136) (JCPDS N°. 21-1276). P25 exhibited more intense and narrower peaks, suggesting a higher degree of crystallinity and a larger crystallite size. Specifically, the crystallite size calculated from the Scherrer equation^22^ for the synthesized TiO_2_NP was approximately 4.00 nm, while the P25 had a crystallite size of 26.0 nm. The smaller crystallite sizes in our study are attributed to the controlled hydrolysis of titanium(IV) isopropoxide. This method allows for the precise regulation of the nucleation and growth phases of TiO_2_ crystals, effectively leading to the production of smaller crystallites.

Figure 1E also displays the Raman spectra of the synthesized titanium dioxide nanoparticles (TiO_2_NPs) and commercial P25 samples. In the TiO_2_NP sample, distinct bands associated with the anatase phase (151, 400, 520, and 636 cm^–1^), denoted as A, are observed. In turn, P25 exhibits these anatase phase bands and two additional bands at 142 and 445 cm^–1^, corresponding to the rutile phase (identified as R).^4,23^ This observation aligns with the results obtained from XRD analysis.

**Figure 2:**
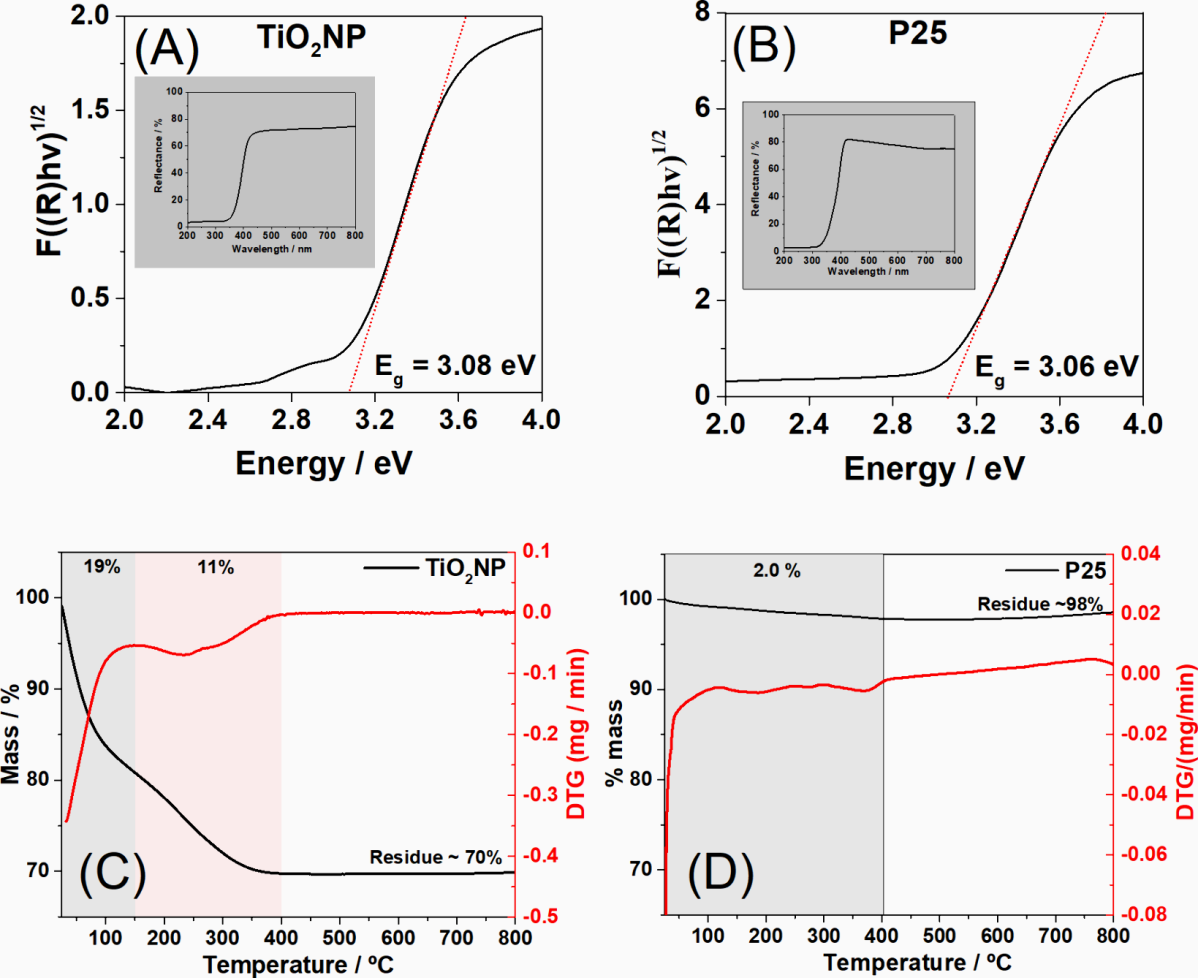
Tauc plot graphs and DRS (inset) of TiO_2_NP (A) and P25 (B) TG and DTG curves of TiO_2_NP (C) and P25 (D).

The bandgap of the TiO_2_NP and P25 samples was assessed using data from the DRS (diffuse reflectance spectroscopy) spectra and Tauc plots (Figures 2A and 2B). In both samples, there was notable absorption in the ultraviolet region, attributed to charge transfer transitions from the valence band, primarily constituted by the 2p orbitals of oxygen, to the 3d t_2_g orbitals of the Ti^4+^ cation.^24^ The bandgap values were 3.08 eV for the TiO_2_NP sample and 3.06 eV for P25.

The thermogravimetric (TG) curves and their first derivatives (DTG) for the TiO_2_NP and P25 samples can be observed in Figure 2C. In the case of the TiO_2_NP sample, we observe an initial mass loss event occurring within the temperature range of 30–150 °C, accounting for approximately 19%. This loss is attributed to the removal of adsorbed water.^25^ The second thermal event observed between 150 and 400 °C represents an additional 11% mass loss and corresponds to the elimination of nitric acid molecules that were initially present in the sample, as they were used during synthesis for pH adjustment. At 800 °C, around 70% of the remaining material is identified as titanium dioxide.^26^ P25 exhibits a single thermal event occurring between 30 and 400 °C, which is associated with the removal of water molecules and the condensation of the hydroxyl groups present on the surface of TiO_2_. This resulted in the significant detection of a 98% residue.

Figure S2 (Supporting Information) presents the relationship between scattered light intensity and hydrodynamic diameter (*D*_h_) for TiO_2_NP and P25 samples dispersed in water. The average hydrodynamic diameter of the synthesized titanium dioxide nanoparticles measured approximately 73 ± 10 nm, whereas for the commercial P25, it was approximately 152 ± 7 nm. These findings are consistent with the XRD data and align with previously reported research in the literature.^27^ The zeta potential for the P25 dispersion was determined to be 35.2 ± 0.5 mV, which aligns with previously reported data in the literature.^28^ For the synthesized titanium dioxide nanoparticles, the measured zeta potential was 40.6 ± 1.9 mV. These positive values indicate a positively charged surface for the particles in the dispersion. This positive charge results from the particles being prepared at a pH of 1.5, which is below the isoelectric point of titanium dioxide (6.2 for P25) leading to the protonation of surface hydroxyl groups (Figure S1, Supporting Information).^29^ It is worth noting that electrostatically stabilized nanoparticle dispersions are typically considered stable when they have zeta potential values above +30 mV or below −30 mV.^30^ As such, both the dispersions containing P25 and those with TiO_2_NP produced in an aqueous medium are considered to be stable.

**Figure 3:**
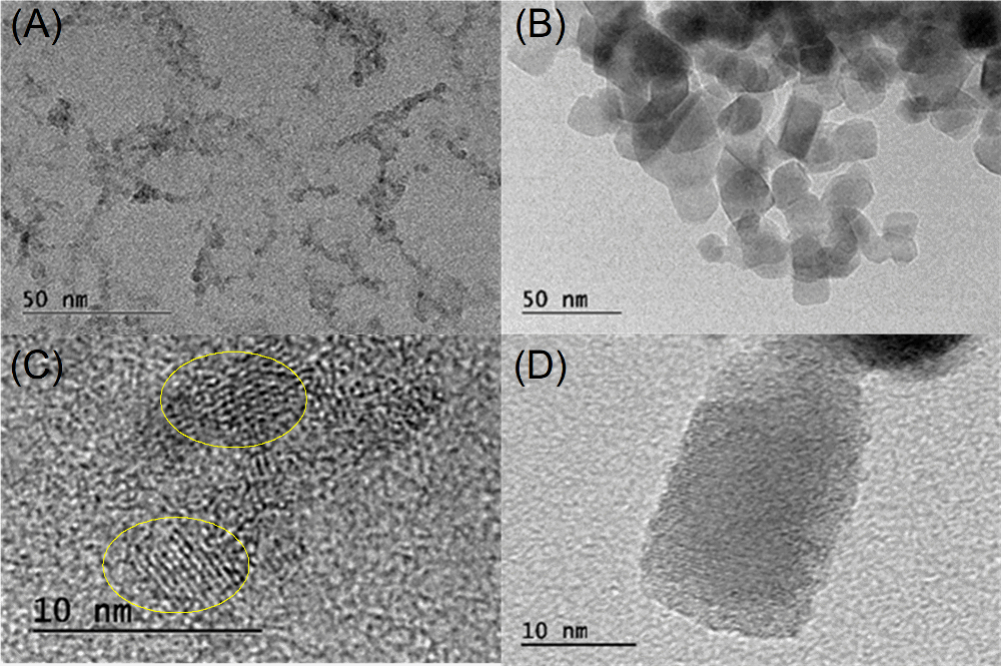
TEM images of the TiO_2_NP (A, C) and P25 (B, D).

TiO_2_NP particles are smaller than 10 nm and appear to be more tightly grouped than those in the P25 sample (Figure 3A,D). P25 particles are roughly 30 nm in size, according to the transmission electron microscopy images (Figure 3B). Crystalline fringes (highlighted by yellow circles) with an interplanar distance of 0.35 nm, which corresponds to the (101) plane, are indicative of the exclusive presence of the anatase phase in this sample (Figure 3C).^31^ The BET (Brunauer, Emmett, and Teller) surface areas of these samples were determined through nitrogen desorption–adsorption analysis (BET). The nanoparticles prepared in this study exhibit a surface area of 86.373 m^2^.g^–1^, whereas the commercial P25 has a surface area of 41.955 m^2^.g^–1^.

**Figure 4:**
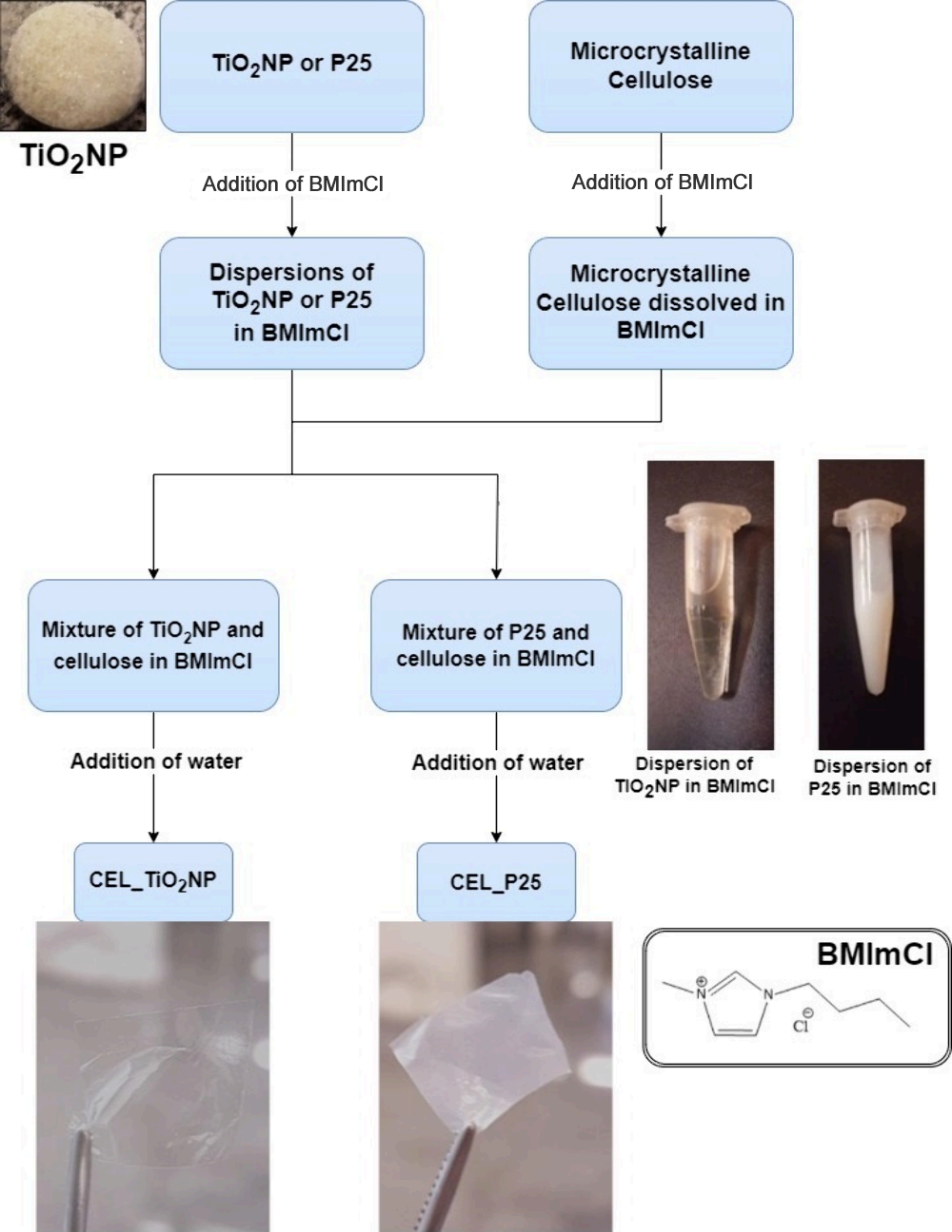
Flowchart of the preparation of the membranes.

To prepare the membranes, we used a formulation containing 20% TiO_2_NP or P25 by mass relative to the cellulose mass. The detailed process flowchart is in Figure 4. Initially, we prepared individual dispersions of TiO_2_NP and P25 in BMImCl. Figure 4 illustrates the distinct visual feature of these dispersions: the TiO_2_NP dispersion appears translucent and colorless, while that of P25 is opaque and white. The difference in opacity is primarily due to the size of the nanoparticles; larger particles scatter light more effectively, resulting in increased opacity. Following this, each dispersion was individually blended with a cellulose solution in BMImCl. We chose this method to improve the compatibility between the inorganic and organic components of the membranes, facilitated by their mutual solubility/dispersity in BMImCl. The interaction between 1-butyl-3-methylimidazolium chloride (BMImCl) and cellulose, which is responsible for the dissolution of the biopolymer, is illustrated in Figure S3. After blending, the solutions were spread out using a spin coater, and the addition of water caused membrane formation. The resulting membranes were then thoroughly washed and dried. Figure 4 shows the CEL_TiO_2_NP, notable for its translucency, alongside CEL_P25, which exhibits an opaque appearance.

**Figure 5:**
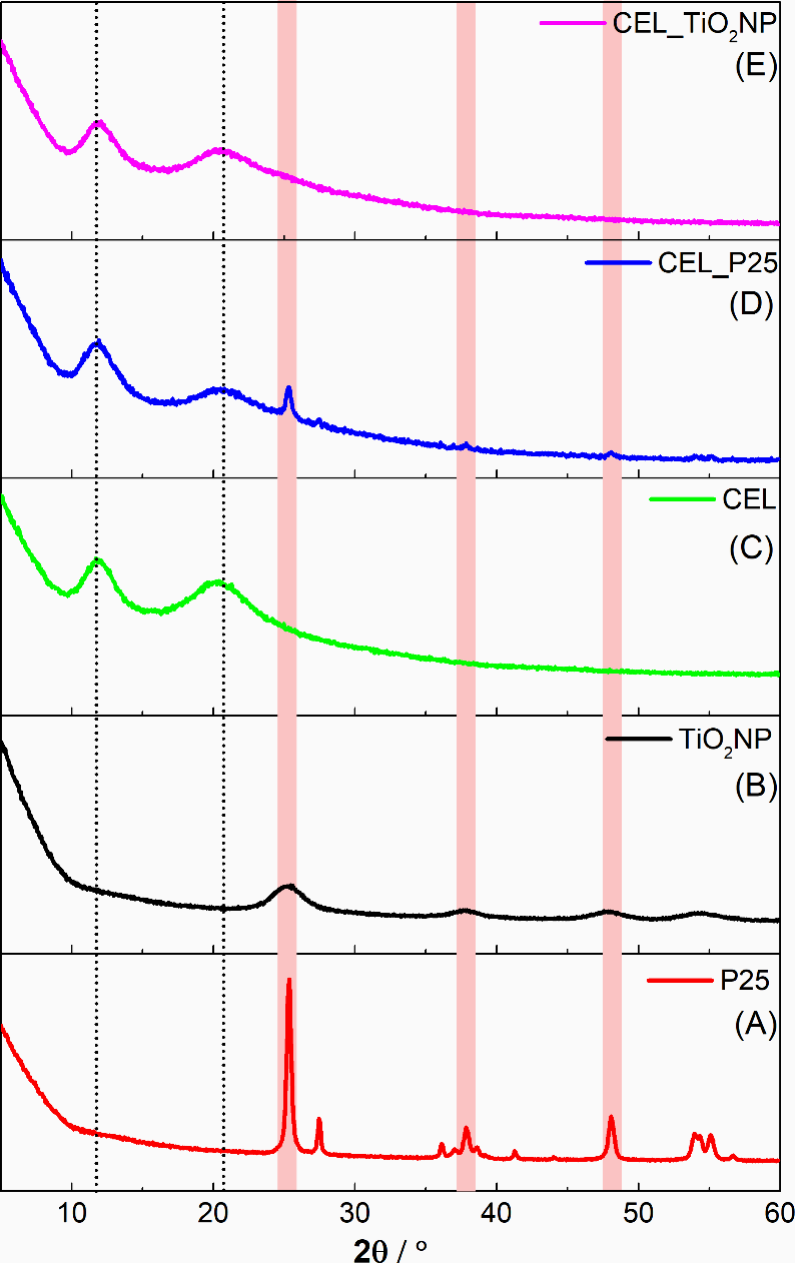
X-ray diffractograms of P25 (A), TiO_2_NP (B), cellulose (C), CEL_P25 (D), and Cel_TiO_2_NP (E).

Figure 5 displays the X-ray diffractograms of cellulose films embedded with P25 and TiO_2_NP nanoparticles compared with that of pure cellulose. As the diffractogram of pure cellulose, the membranes containing the inorganic particles show two low-intensity reflections at 2θ = 11.8 and 20.5°, characteristic of type II cellulose, as indicated by dotted lines.^32^ The diffractogram of cellulose with P25 reveals an additional peak at 2θ = 25.3° from the crystallographic planes (101) of TiO_2_ in the anatase phase. On the other hand, the diffractogram of the cellulose membrane containing TiO_2_NP does not exhibit any new reflections. This is likely due to the nanometer size of the TiO_2_NP crystallites, leading to their extensive dispersion within the polymer matrix.

**Figure 6:**
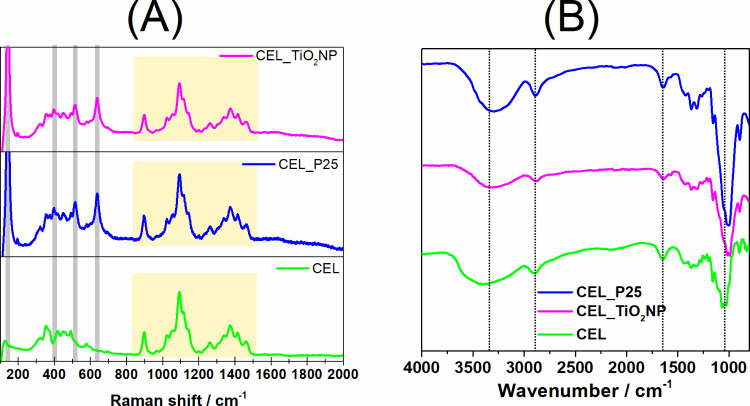
Raman (A) and FTIR (B) spectra of the cellulose (green), CEL_P25 (blue), and CEL_TiO_2_NP (magenta) membranes.

Raman spectroscopy played a crucial role in verifying the presence of TiO_2_ in the cellulose membranes. As can be seen in Figure 6A, the spectra of CEL_TiO_2_NP (magenta curve) and CEL_P25 (blue curve) display four principal bands at approximately 142, 400, 520, and 636 cm^–1^ (highlighted with gray rectangles), which are indicative of TiO_2_. The pure cellulose membrane (green curve) exhibits several bands between 850 and 1500 cm^–1^ (highlighted with a yellow rectangle), corresponding to the vibrations of functional groups in cellulose, such as O–H, C–O–C, C–O, etc.^33^ These bands remain unchanged when TiO_2_ is added to the cellulose membranes, indicating that the primary chemical structure of the biopolymer is maintained. The FTIR spectra, as shown in Figure 6B, also confirm the preservation of the primary structure of cellulose in the CEL_TiO_2_NP and CEL_P25 membranes. These spectra closely resemble those of the pure cellulose membrane. The main vibrations include the stretching and angular deformation of hydroxyl groups around 3350 and 1650 cm^–1^, respectively. Additionally, the C–H stretching bands are prominent in the 2850–2900 cm^–1^ range. The most pronounced band, found between 1000 and 1200 cm^–1^, is associated with axial deformation of the C–O bond. These characteristic bands align with those of type II cellulose, typically produced by dissolving microcrystalline cellulose and regenerating it using an antisolvent.^34^

**Figure 7:**
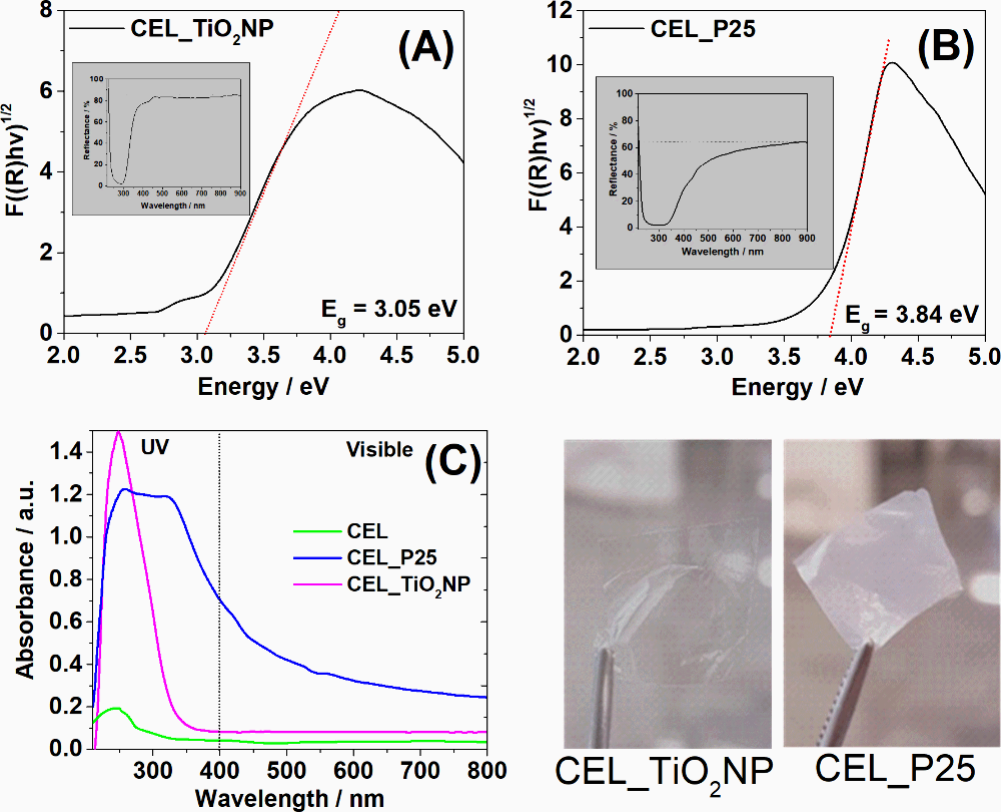
Tauc plot graphs and DRS (inset) of CEL_TiO_2_NP (A) and CEL_P25 (B); UV–vis of CEL (green), CEL_P25 (blue), and CEL_TiO_2_NP (magenta) (C); and photos of the membranes (D).

Figure 7A,B displays the diffuse reflectance spectra of the CEL_P25 and CEL_TiO_2_NP membranes along with the bandgap values determined from Tauc plots. CEL_TiO_2_NP membrane exhibits a bandgap of 3.05 eV, while the CEL_P25 shows a higher bandgap of 3.84 eV. Particularly, the bandgap found for CEL_P25 is higher compared to the bandgap of P25 in the powder form. The variation in band gap behavior between P25 and TiO_2_NP when immobilized on cellulose can be attributed to their composition and particle size. P25, which contains both anatase and rutile phases, is more prone to changes in the electronic structure upon immobilization.^35^ On the other hand, TiO_2_NP, composed entirely of the anatase phase, maintains its band gap when immobilized due to the longer electron–hole pair lifetimes and stability of anatase in sizes below 10–20 nm.^36−38^ In addition, the better dispersion of TiO_2_NP in the cellulose matrix prevents aggregation, thus maintaining its band gap.

Further analysis of the cellulose membranes containing titanium dioxide nanoparticles was performed by using UV–vis absorption spectroscopy, as shown in Figure 7C. The cellulose membrane does not exhibit absorption bands in the visible region (400–800 nm), reflecting its transparent and colorless nature. However, low-intensity bands in the UV (200–300 nm) are seen; these are caused by chromophores (O–H, C–O) found in the cellulose structure.^39^ On the other hand, the presence of titanium dioxide in CEL_TiO_2_NP and CEL_P25 membranes results in an intense and broad absorption band in the UV region (200 to 350 nm). The absence of bands in the visible region confirms the transparency of the CEL_TiO_2_NP membrane. On the other hand, the higher baseline in the visible region is consistent with the opacity of the CEL_P25 film.

**Figure 8:**
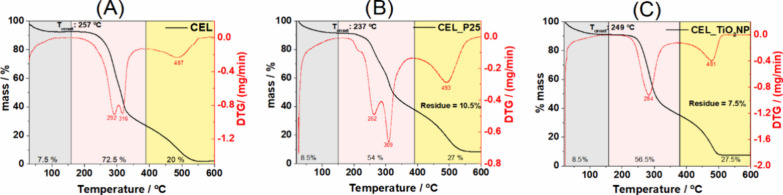
TG and DTG curves of cellulose (A), CEL_P25 (B), and CEL_TiO_2_NP (C).

The thermogravimetric analysis curve of the pure cellulose membrane is shown in Figure 8, where four different events of mass loss are visible. The first stage, which occurs between 25 and 150 °C (7.5 wt % mass loss), is caused by the loss of water in the membrane. The decomposition of cellulose in its more amorphous regions can be seen in the following stages, which can be detected by DTG peaks at 292 and 316 °C. The decomposition of the crystalline regions of the cellulose corresponds to the last stage, which is denoted by a DTG peak at 487 °C.^40^ The cellulose membrane containing P25 (Figure 8B) and TiO_2_NP (Figure 8C) thermogravimetric curves show that the onset temperatures of degradation are 249 and 237 °C, respectively, compared to 257 °C for pure cellulose. This suggests that the addition of TiO_2_ reduces the thermal stability of the polymer, most likely because of the catalytic action of the titanium oxide on the oxidative thermal degradation of cellulose.^41−43^ Despite this, the overall high thermal stability makes these membranes suitable for use in heterogeneous catalysts. Because titanium dioxide is hygroscopic, the amount of adsorbed water (the first event observed between 25 and 150 °C) in these samples is slightly higher than in pure cellulose.^44^ Cellulose membrane with P25 yielded a residue of 10.5 wt %, whereas the sample CEL_TiO_2_NP produced a residue of 7.5 wt %. Considering that the theoretical residue for the CEL_P25 would be roughly 15 wt % and for the CEL_TiO_2_NP sample 11 wt %, we can infer that some of the TiO_2_ nanoparticles were lost during the film-making process. This phenomenon was also observed in other studies involving the production of cellulose membranes.^45^

The Ti content in the samples was determined by using the ICP-OES technique. The CEL_TiO_2_NP sample shows approximately 2.93 wt % titanium, which corresponds to about 7.50% titanium dioxide. Meanwhile, CEL_P25 exhibited 4.06 wt % of Ti, equivalent to approximately 10.0% titanium dioxide. These findings are consistent with the thermogravimetric analysis results.

**Figure 9:**
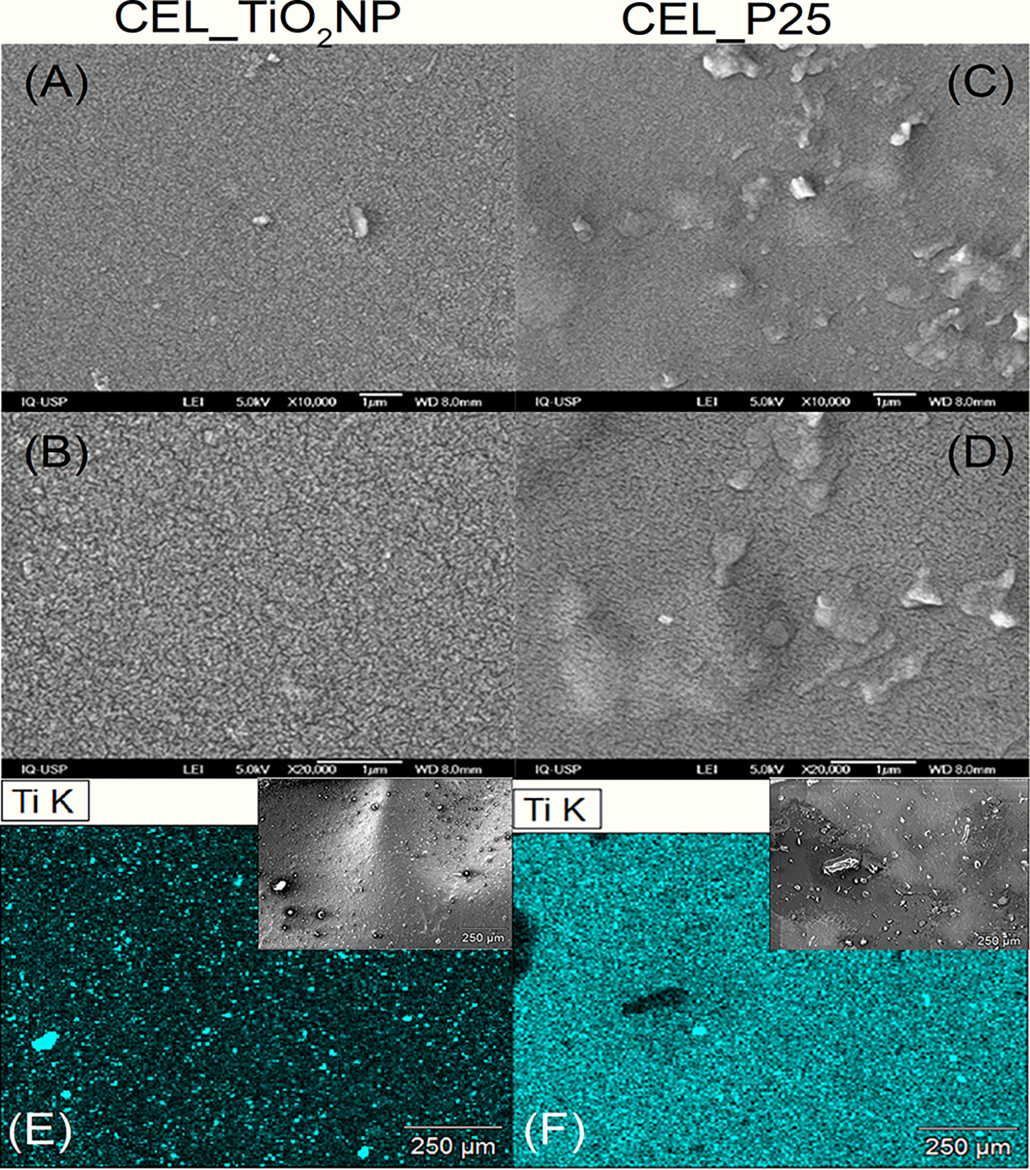
SEM and EDS mapping analysis of CEL_TiO_2_NP (A, B, and E) and CEL_P25 (C, D, and F).

SEM images of cellulose membranes embedded with TiO_2_NP (Figure 9A,C) and P25 (Figure 9B,D) at different magnifications are displayed in Figure 9. Comparing the cellulose membrane with TiO_2_NP nanoparticles to the CEL_P25 sample, the former exhibits a smoother and more uniform surface, while the latter has a rougher surface with noticeable clusters. Using energy-dispersive X-ray spectroscopy (EDS) mapping analysis, the uniform distribution of titanium atoms within these samples was determined (Figure 9E,F). This analysis highlights the effectiveness of the synthesis method used by confirming that the TiO_2_ particles are uniformly distributed throughout the cellulose membrane with no segregated phases.

The photocatalytic tests were conducted at pH 2. This choice is based on numerous studies in the literature, including Vautier et al.,^46^ which demonstrates that the decolorization of Indigo Carmine (IC) using metal oxides is more efficient at this pH level. The reason for this efficiency is that, at lower pH values, the surface of the metal oxide particles becomes positively charged. This leads to more favorable electrostatic interactions between the catalyst and anionic dye molecules.

**Figure 10:**
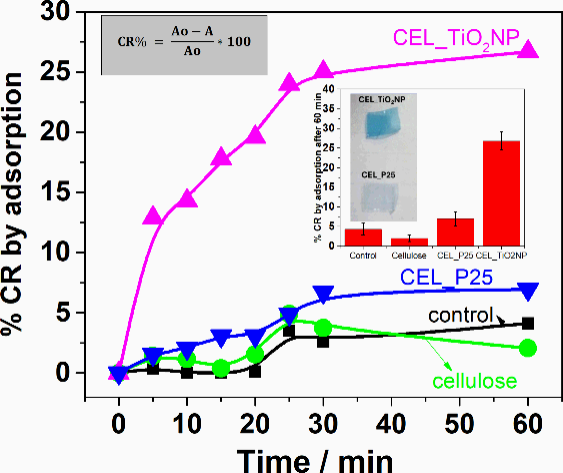
%CR by adsorption over time for the control experiments and those containing cellulose membranes. Inset: %CR after 60 min and photographs of the membranes after the adsorption experiment.

The initial phase of the study involved assessing the adsorption capacity of the membranes. This was done in darkness over a 60 min period, during which aliquots were taken at predetermined intervals to measure the absorption band at 610 nm (Figure 10). It was observed that after 30 min, the absorption values stabilized, suggesting that the adsorption process had reached equilibrium. The percentage of color removal (%CR) due to adsorption over time was calculated using the formula presented in the inset of Figure 10. For comparative purposes, two experiments were conducted: one with a pure cellulose membrane and the other without any membrane. After 60 min (inset Figure 10), the percentage of color removal obtained in the test using pure cellulose was almost the same as it was in the control experiment, with an approximate percentage of 4%. On the other hand, this percentage for the titanium dioxide-containing cellulose membranes was 27% for the CEL_TiO_2_NP sample and 7% for the CEL_P25 membrane. The photographs of the CEL_TiO_2_NP and CEL_P25 membranes after the adsorption process are presented in the inset of Figure 10. The more intense blue color observed in the CEL_TiO_2_NP membrane correlates with a higher extent of dye adsorption. These images were included to visually demonstrate the efficacy of the photocatalytic activity and the extent of dye removal achieved by each type of membrane. The enhanced adsorption observed in the CEL_TiO_2_NP sample compared to CEL_P25 was attributed to the unique properties of the TiO_2_ nanoparticles embedded within the cellulose matrix. The smaller size and higher surface area of these nanoparticles, relative to those in the CEL_P25 sample, increase the availability of functional groups, such as hydroxyl groups. These groups effectively interact with Indigo Carmine molecules through hydrogen bonding—a mechanism also observed in other oxides like silica and manganese oxide, where adsorbent groups containing hydroxyl groups form hydrogen bonds with the hydroxyl and amine groups of dyes.^47−49^ Furthermore, due to their smaller size, the TiO_2_ nanoparticles are more uniformly dispersed within the cellulose matrix compared to those in the CEL_P25 sample. This uniform dispersion prevents the agglomeration of TiO_2_ particles, thereby maintaining their high surface area and ensuring greater accessibility for dye molecules, which significantly enhances the adsorption performance.

**Figure 11:**
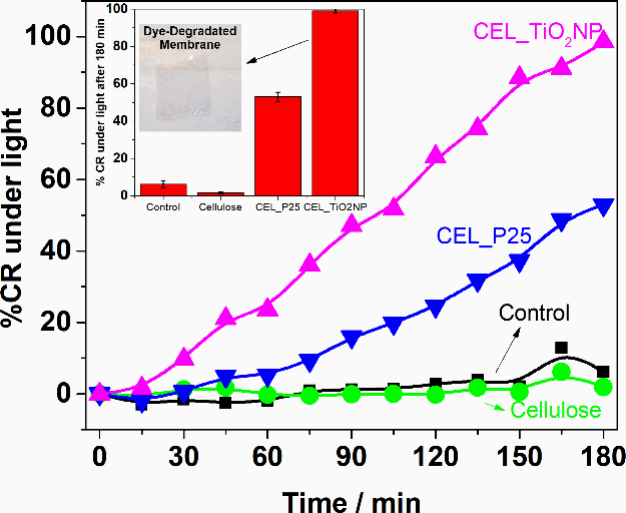
%CR under light over time for the control experiments and those containing a cellulose membrane. Inset: %CR under light after 180 min and the photograph of CEL_TiO_2_NP membrane after the discoloration experiment.

The discoloration of the IC under illumination was observed over 180 min. At 15 min intervals, aliquots were sampled. The UV–vis spectra of these aliquots were recorded (Figure S4), and the equation in the inset of Figure 10 was used to calculate the color removal (% CR) caused by photocatalysis. In the absence of a catalyst, there was no noticeable change in the absorbance, indicating negligible self-degradation. Membranes containing P25 and TiO_2_NP exhibited 53 and 100% discoloration, respectively, after 180 min, whereas the cellulose membrane demonstrated no activity. The CEL_TiO_2_NP membrane became colorless after this process, as shown in the inset of Figure 11, indicating the degradation of the adsorbed dye.

To assess the possible degradation products formed, we further evaluate the UV–vis of the solutions after the discoloration experiments. Figure S5 shows the UV–vis absorption bands of Indigo Carmine solutions before and after complete discoloration, following 180 min of contact with the CEL_TiO_2_ sample. In the Indigo Carmine spectrum, the absorption peaks at 285 and 610 nm are related to the cross-conjugated system in the dye molecule. After complete discoloration, these two bands disappear completely, indicating the degradation of Indigo Carmine. The product formed was attributed to isatin 5-sulfonic acid (inset, Figure S5), derived from the homolytic cleavage of the dye, supported by the presence of an absorption band at 240 nm. The absence of additional peaks in the UV–vis analysis suggests that there is no significant formation of complex byproducts.^46,50,51^

By plotting ln(*A*/*A*_o_) with time, we assessed the kinetics. The nonlinear trends observed in these plots (Figure S6, Supporting Information) suggest that the reactions do not adhere to first-order kinetics, which is typically observed with powder TiO_2_. Several factors might explain these observations: (a) The immobilization of TiO_2_ on cellulose membranes could create diffusion barriers. Reactants and products need to diffuse through the cellulose matrix to reach the TiO_2_ active sites and then diffuse back into the bulk solution. This slower diffusion process compared to the surface reaction can lead to mass transfer limitations, deviating from first-order kinetics. (b) The presence of a cellulose membrane might influence the penetration and distribution of light, crucial for photocatalysis. If the light distribution is uneven or if the membrane absorbs or scatters light, it can lead to nonuniform activation of TiO_2_, resulting in nonfirst-order kinetics.

It is worth noting that when bare TiO_2_NP and P25 were used as catalysts, the discoloration of the IC caused by photocatalysis was achieved in less than 25 min for both catalysts (Figure S7, Supporting Information). This faster discoloration, compared with the membranes, is attributed to the higher surface area of these powders. However, despite this enhanced performance, practical application was hindered by the difficulty of recovering and reusing the catalysts. The lack of a straightforward recovery process rendered the reuse of both TiO_2_NP and P25 catalysts impractical in this context.

For the reuse tests, the CEL_TiO_2_NP membrane was subjected to three cycles to assess its efficiency and durability. The performance across these cycles was assessed by a specific metric for comparative analysis, the turnover frequency (TOF) value. This metric involved calculating the ratio of the mass of Indigo Carmine (IC) degraded to the mass of TiO_2_, adjusted for the same units, and then normalizing this by the time (in hours) taken to achieve 99% decolorization. The calculated values for the first, second, and third cycles were 0.09 ± 0.01, 0.07 ± 0.02, and 0.08 ± 0.02 h^–1^, respectively. These results suggest that the CEL_TiO_2_NP membrane maintains its catalytic efficiency consistently across multiple cycles without notable degradation in the performance. To further demonstrate the stability of the CEL_TiO_2_NP sample after photocatalysis, additional experiments were performed, including XRD and FTIR analyses on the samples after multiple cycles (Figure S8, Supporting Information). The analyses confirm that there are no significant changes in its crystalline structure or chemical composition after repeated use, corroborating its stability and durability.

## 4 Conclusions

In conclusion, the successful synthesis of titanium dioxide nanoparticles in the anatase phase, which is known for its superior photocatalytic capabilities, was achieved. These nanoparticles differed markedly from commercially available P25 in terms of crystallinity, particle size, and optical characteristics. The incorporation of these nanoparticles into cellulose membranes resulted in notable differences in the physical and chemical properties of the resultant composites. The cellulose membrane embedded with TiO_2_NP (CEL_TiO_2_NP) exhibited enhanced adsorption capacity and photocatalytic efficiency against Indigo Carmine dye, surpassing those of the P25 counterparts. This improvement can likely be attributed to the larger surface area and greater reactivity of the synthesized TiO_2_NPs. Additionally, the CEL_TiO_2_NP membrane displayed stability and reusability, consistently maintaining its catalytic efficiency across multiple cycles without significant degradation. Notably, the use of these membranes in heterogeneous catalysis emphasizes the advantages in terms of ease of isolation and reuse of the catalyst, which is pivotal for practical applications. Thus, this study confirms that employing synthesized TiO_2_NPs in cellulose membranes is an effective strategy for enhancing both photocatalytic and adsorptive properties, paving the way for the development of efficient, reusable photocatalytic materials.

## Supporting Information Available

Schematic diagrams illustrating the photocatalytic degradation mechanism of TiO_2_, scattered light intensity data for TiO_2_NP and P25 samples dispersed in water, illustrations of interactions between BMImCl, cellulose, and TiO_2_, UV–vis spectra showing Indigo Carmine decolorization under UV light, kinetic treatment plots, % color removal data for IC under different conditions, and XRD and FTIR analysis of the CEL_TiO_2_NP sample before and after multiple cycles of photocatalysis (PDF)
