# Supplementary material for: Eco-Friendly Photocatalytic Solutions: Synthesized TiO2 Nanoparticles in Cellulose Membranes for Enhanced Degradation of Indigo Carmine Dye
Source: ACS Omega. 2024 Oct 15;9(43):43395–405. doi: 10.1021/acsomega.4c04017 (PMC11525746; doi:10.1021/acsomega.4c04017)
Supplement: Supplementary file 1 — ao4c04017_si_001.pdf [file ao4c04017_si_001.pdf]

Supporting Information

Eco-Friendly Photocatalytic Solutions: Synthesized TiO_2 Nanoparticles in Cellulose Membranes for Enhanced Degradation of Indigo Carmine dye

Arthur Matsudo[1], Larissa V. F. Oliveira[2], Tereza S. Martins,[1] Fernanda F. Camilo[1*]

[1]Chemistry Department, Institute of Environmental, Chemical and Pharmaceutical Sciences, Federal University of São Paulo, SP-09913-030, Diadema, Brazil

[2]Center of Natural Sciences and Humanities, Federal University of ABC, SP-09210-580, Santo Andre, Brazil

*corresponding author

Email: ffcamilo@unifesp.br

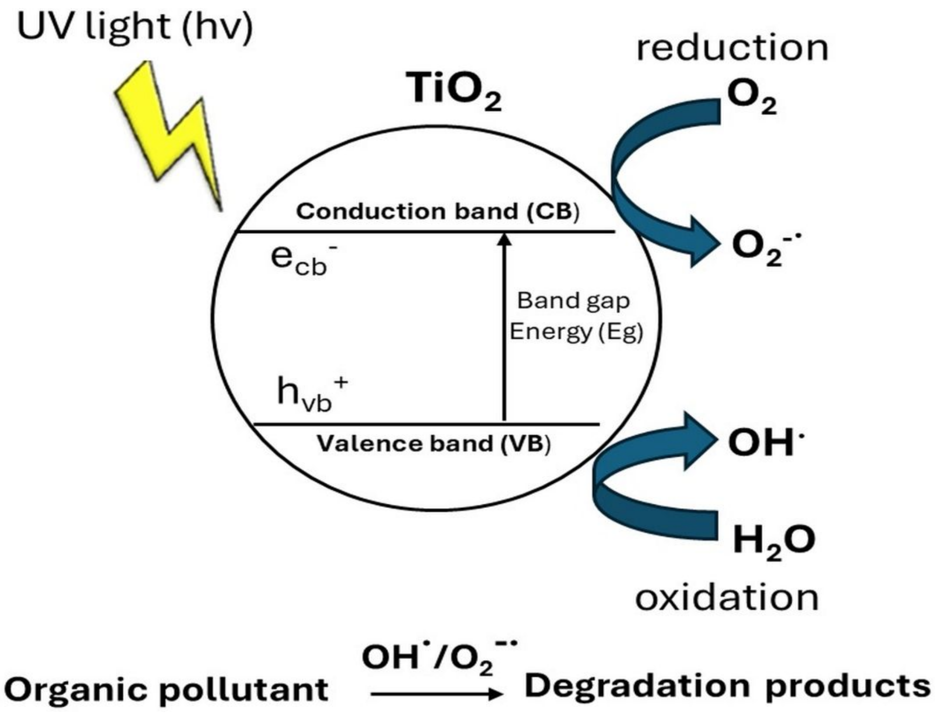

Figure S1: Schematic diagram illustrating the photocatalytic degradation mechanism using TiO_2

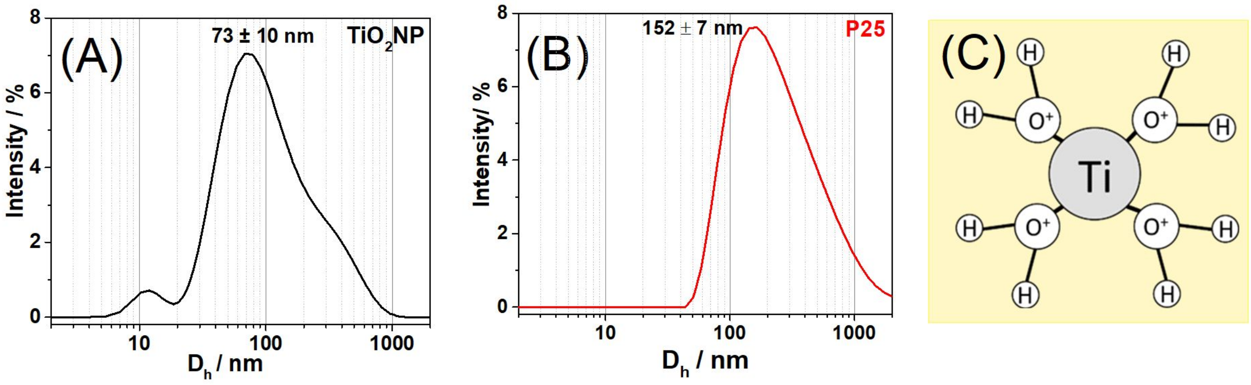

Figure S2: Scattered light intensity vs. hydrodynamic diameter (Dh) for TiO_2NP (A) and P25 (B) samples dispersed in water and an illustrative scheme of the protonated OH groups of TiO_2 at pH 1.5 (C).

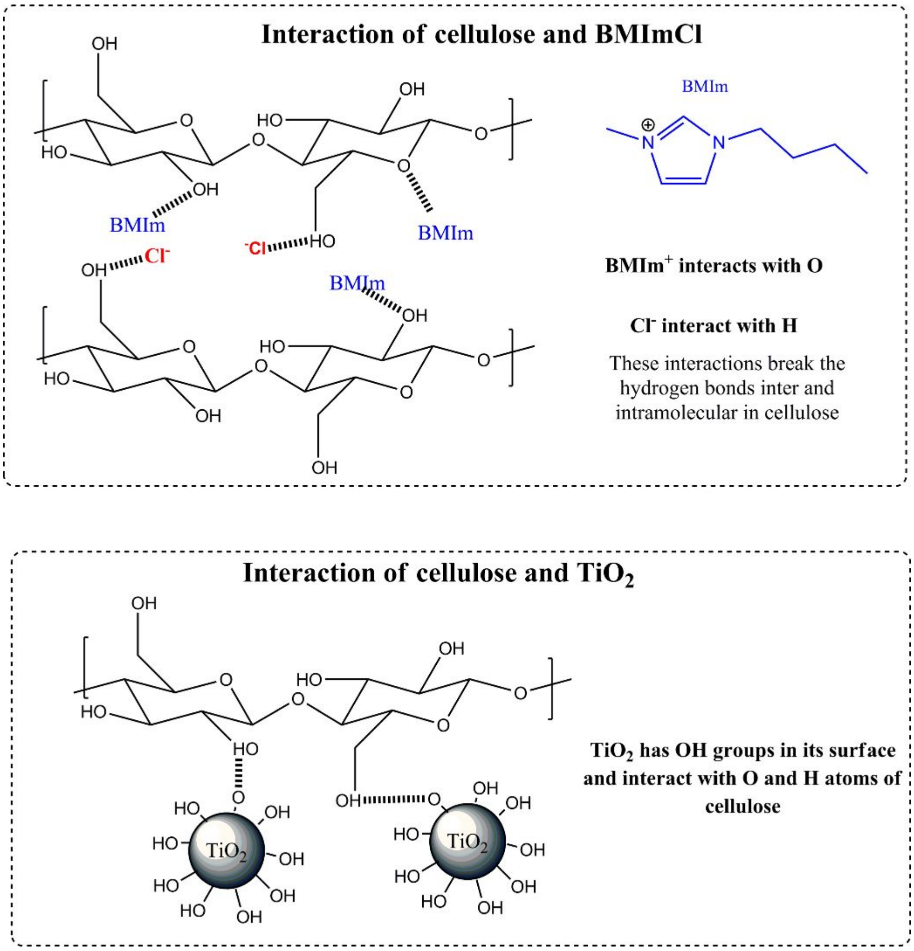

Figure S3: Illustration of interaction between BMImCl and cellulose and TiO_2 and cellulose

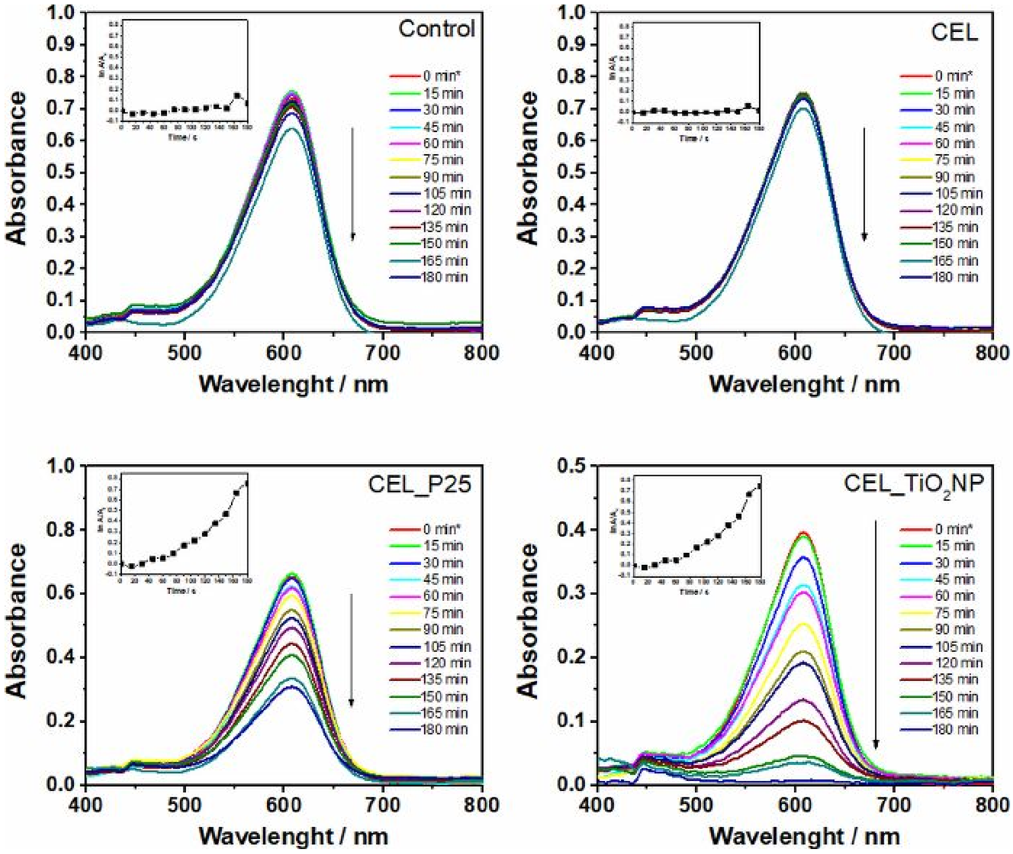

Figure S4: UV-Vis spectra of aliquots of reaction media during Indigo Carmine decolorization tests under UV light

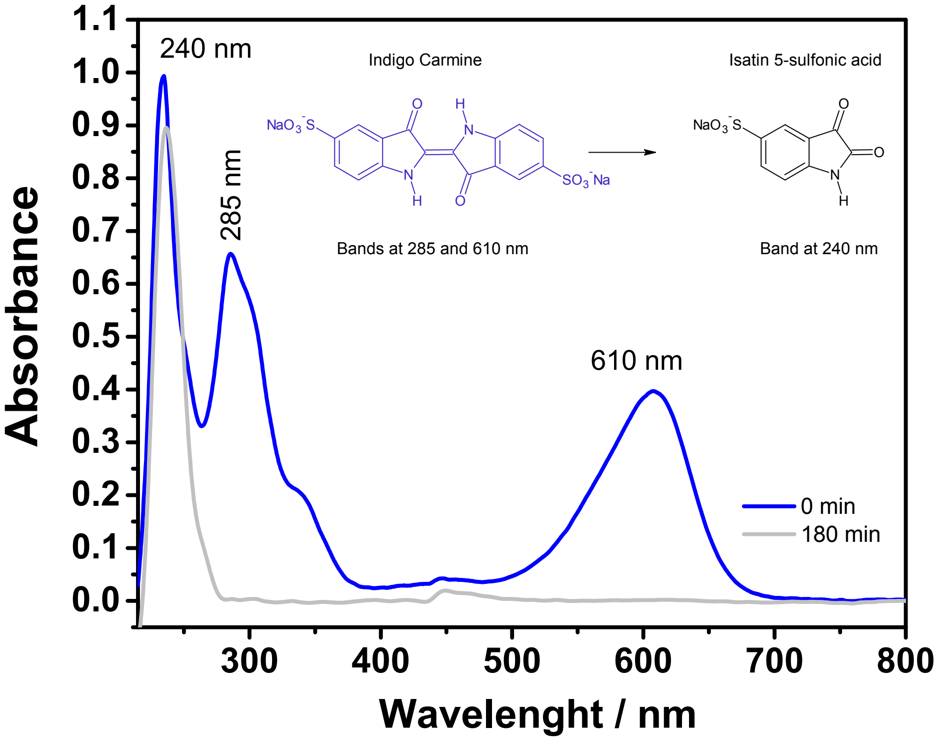

Figure S5: UV-Vis spectra of indigo carmine solutions before and after complete discoloration using CEL_TiO_2NP as a catalyst

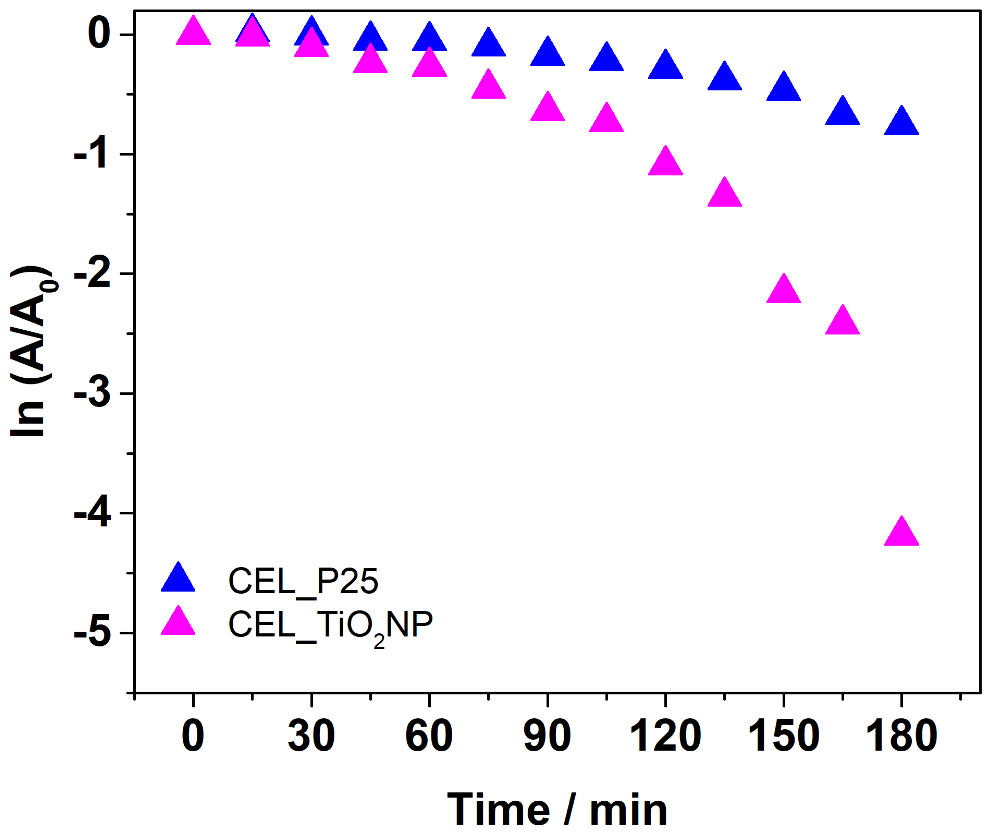

Figure S6: Kinetics treatment - ln(A/A_0) versus time.

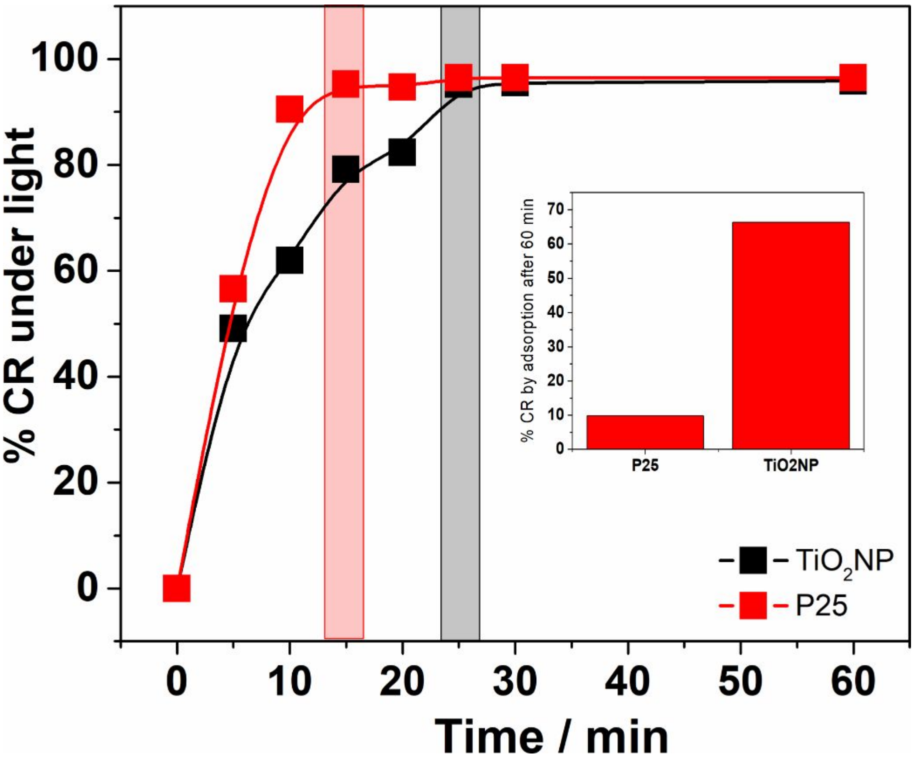

Figure S7: % Color Removal of IC under light using bare-TiO_2NP and P25. Inset %CR under dark after 60 min.

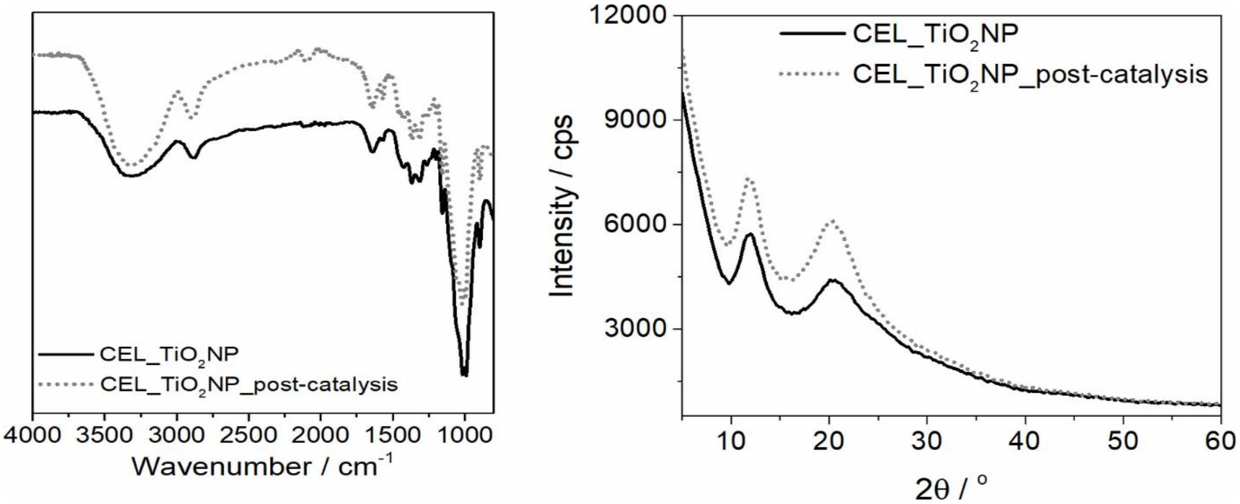

Figure S8: XRD and FTIR analysis of the CEL_TiO_2NP sample before and after multiple cycles of photocatalysis
